# Wuhan to World: The COVID-19 Pandemic

**DOI:** 10.3389/fcimb.2021.596201

**Published:** 2021-03-30

**Authors:** Ashok Kumar, Rita Singh, Jaskaran Kaur, Sweta Pandey, Vinita Sharma, Lovnish Thakur, Sangeeta Sati, Shailendra Mani, Shailendra Asthana, Tarun Kumar Sharma, Susmita Chaudhuri, Sankar Bhattacharyya, Niraj Kumar

**Affiliations:** ^1^Translational Health Science and Technology Institute (THSTI), Faridabad, India; ^2^Manipal Academy of Higher Education, Manipal, India; ^3^Jawaharlal Nehru University, New Delhi, India; ^4^Central University of Haryana, Mahendragarh, India

**Keywords:** SARS-CoV-2, COVID-19, coronaviruses, transmission, diagnostics, therapeutics

## Abstract

COVID-19 is a Severe Acute Respiratory Syndrome (SARS), caused by SARS-CoV-2, a novel virus which belongs to the family *Coronaviridae*. It was first reported in December 2019 in the Wuhan city of China and soon after, the virus and hence the disease got spread to the entire world. As of February 26, 2021, SARS-CoV-2 has infected ~112.20 million people and caused ~2.49 million deaths across the globe. Although the case fatality rate among SARS-CoV-2 patient is lower (~2.15%) than its earlier relatives, SARS-CoV (~9.5%) and MERS-CoV (~34.4%), the SARS-CoV-2 has been observed to be more infectious and caused higher morbidity and mortality worldwide. As of now, only the knowledge regarding potential transmission routes and the rapidly developed diagnostics has been guiding the world for managing the disease indicating an immediate need for a detailed understanding of the pathogen and the disease-biology. Over a very short period of time, researchers have generated a lot of information in unprecedented ways in the key areas, including viral entry into the host, dominant mutation, potential transmission routes, diagnostic targets and their detection assays, potential therapeutic targets and drug molecules for inhibiting viral entry and/or its replication in the host including cross-neutralizing antibodies and vaccine candidates that could help us to combat the ongoing COVID-19 pandemic. In the current review, we have summarized the available knowledge about the pathogen and the disease, COVID-19. We believe that this readily available knowledge base would serve as a valuable resource to the scientific and clinical community and may help in faster development of the solution to combat the disease.

## Coronaviruses

**Figure 1:**
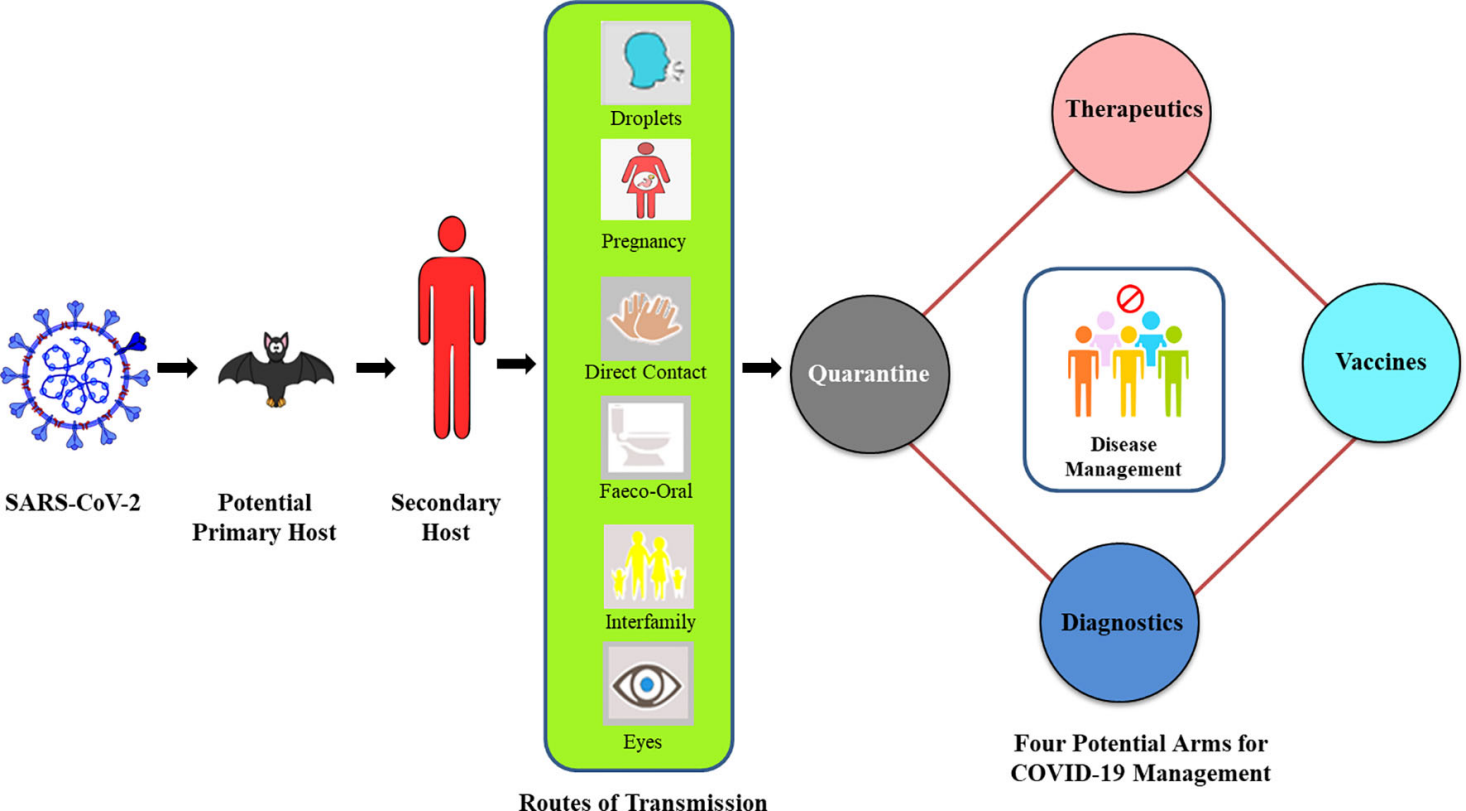
Overview of COVID-19 progression and key four-arms for its management.

Coronaviruses are roughly spherical enveloped RNA viruses that belong to the *Coronaviridae* family under the order *Nidovirales* (Payne, 2017). The average diameter of virion particles is in the range of 120–160 nm (Schoeman and Fielding, 2019). The virion particles are typically decorated with petal shape projections (Spike proteins) and based on the crown-like appearance under an electron microscope, the virus was named “Corona” virus (Korsman et al., 2012). As compared to the other RNA viruses, coronaviruses characteristically have a very large genome (~28–32 kb) (Pal et al., 2020). To date, a variety of animals apart from humans (including pigs, cats, rats, cows, bats, pigeons, and ducks) have been serving as a host for coronaviruses with the principal clinical symptom(s) being the respiratory or enteric diseases (Banerjee et al., 2019) (**Figure 1**). The coronaviruses have a long history, however, published reports of their existence are available since the 1960s onwards only (Cui et al., 2019). The coronaviruses majorly came under the focus only after the sudden outbreak of Severe Acute Respiratory Syndrome (SARS) in 2002–2003 in the Guangdong, southern province of China. The members of the coronavirus family are subdivided into three groups, almost all members of group 1 and group 2 viruses have mammalian hosts whereas, in contrast, group 3 coronaviruses have specifically been isolated from birds only (Masters, 2006). SARS-CoV belongs to group 2. As of now, a total of seven coronavirus strains, HCoV-229E, HCoV-NK63, HCoV-OC43, HCoV-HKU1, SARS-CoV, MERS-CoV, and SARS-CoV-2 that crossed the species barriers and infect humans, have been identified (Liu et al., 2020a). Of these, only SARS-CoV, MERS-CoV, and SARS-CoV-2 were able to cause the deadly disease in the humans.

## Emergence of COVID-19

In December 2019, some of the local hospitals in the Wuhan city of China reported several patients with atypical pneumonia of unknown cause (She et al., 2020). Interestingly, most of the patients were linked with the Huanan Seafood Wholesale market in Jianghan District, Wuhan. Considering the gravity of the situation, the Chinese Government declared a public health emergency and formal investigation of the matter on December 31, 2019, with the National Health Commission (NHC, China), Center for Disease Control and Prevention (CDC, China), and Wuhan Municipal Health Commission to find out the cause and the etiological agent (Callaway, 2020). Broncho-alveolar lavage (BAL) samples were collected from patients at Wuhan Jinyintan Hospital on December 30, 2019 and utilized to infect human airway epithelial cells (Vero-E6 and Huh-7) (Lu et al., 2020b). The total RNA was extracted from these infected cells and used to clone and sequence the causative agent(s). Most of sequence reads matched against the lineage B of the genus beta-coronavirus and showed more than 85% identity with bat-SL-CoVZC45 coronavirus and 80% identity to SARS Corona-Virus (SARS-CoV) (Lu et al., 2020b). Therefore, this newly isolated virus was named as novel coronavirus 2019 (2019-nCoV). Considering the degree of homology, the International Committee on Taxonomy of Viruses (ICTV), the global nodal agency holding the responsibility of classification and nomenclature of viruses, renamed the 2019-nCoV as SARS-CoV-2 and, later on, the disease caused by SARS-CoV-2 was announced as COVID-19 by the WHO (Coronaviridae Study Group of the International Committee on Taxonomy of, 2020).

**Figure 2:**
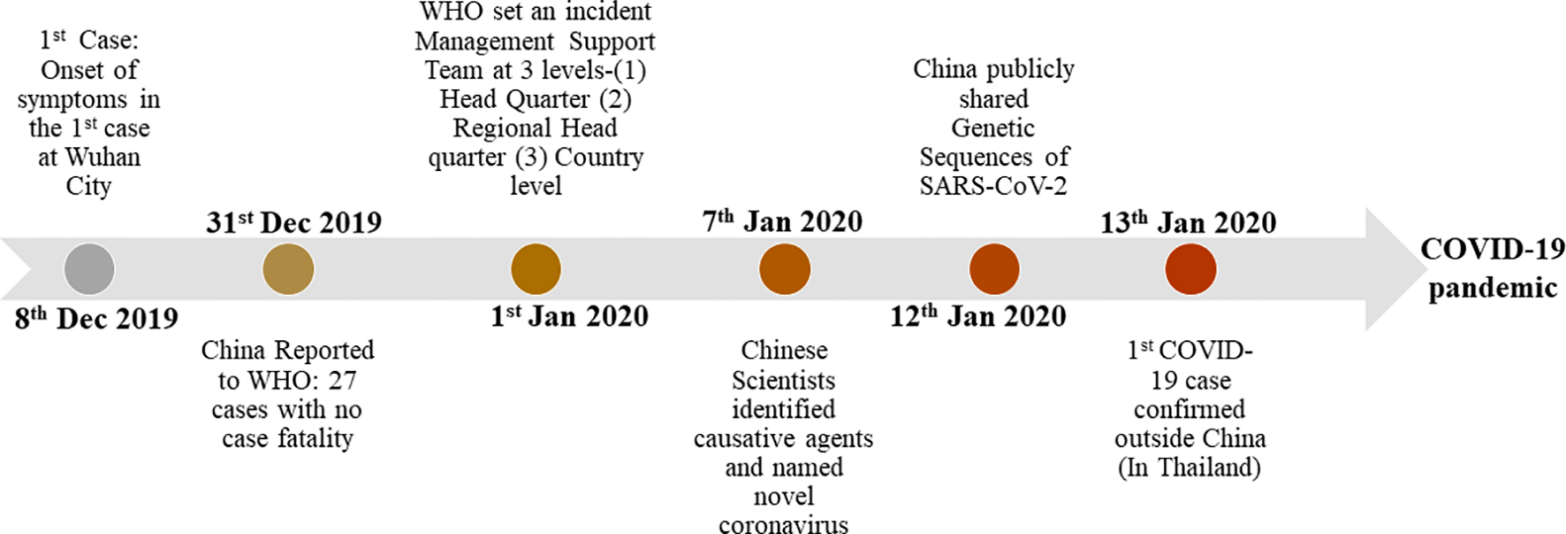
The major chronological events in the emergences of SARS-CoV-2.

As per the report entitled “The Epidemiological Characteristics of an Outbreak of 2019 Novel Coronavirus Diseases (COVID-19-China), 2020” published by the CDC-China on February 11, 2020, a total of 72,314 cases of COVID-19 were recorded. Of these, based on the viral RNA detection in the samples, 62% were classified as confirmed positive; whereas based on the symptoms and exposure, 22% as suspected cases. While 1% of the cases were classified as asymptomatic, meaning that they were diagnosed positive for viral nucleic acid but lack typical symptoms of COVID-19 includes dry cough, tiredness, sore throat and/or shortness of breath, etc. Approximately, ~87% of cases in China belonged to the age group between 30 and 79 years, 3% were 80 years or older, 1% were aged between 10 and 19, years and 1% to the age group of 9 years or younger (Armitage and Nellums, 2020). Majority of these cases (~81%) were classified as mild (either not have any kind of pneumonia or with mild pneumonia), 14% as severe, and 5% as critical. Most of the COVID-19 critical cases were observed to be associated with respiratory failure, septic shock, chest pain, multiple organ dysfunction/failure, and/or loss of speech and movement. The case fatality rate (CFR) of the COVID-19 infection was observed to be ~2.3% (1,023 deaths among 44,672 confirmed cases) (Onder et al., 2020). However, the highest CFR (14.8%) was observed in patients who were aged ≥80 years or who had pre-existing co-morbidities (*i.e.* high blood pressure, cardiovascular disease, diabetes, chronic respiratory issues, and cancer) followed by 8% in patients aged 70–79 years. Interestingly, no case fatality was reported for patient’s aged ≤9 years. Although enormous efforts were made by Chinese health agencies to control the transmission, SARS-CoV-2 got spread across the world in a very short period of time (Guan et al., 2020) (**Figure 2**).

According to the data released by WHO till February 26, 2020 SARS-CoV-2 infected ~112.20 million individuals and caused the death of ~2.49 million individuals across 215 countries of the world (WHO, 2020b). It has severely affected all the continents with the highest in America followed by Europe, Western Pacific, South-East Asia, and Africa (**Table 1**).

**Table 1:** WHO Region wise reported laboratory-confirmed COVID-19 cases, deaths, and case fatality rate (CFR) as of February 26, 2021.

WHO Region	Confirmed COVID-19 cases	Confirmed deaths (COVID-19)	CFR%
AFRO	2,819,018	71,354	2.53
AMRO	49,873,762	1,188,087	2.38
EMRO	6,297,770	143,380	2.27
EURO	38,176,678	852,546	2.23
SEARO	13,440,545	206,820	1.53
WPRO	1,601,297	28,576	1.78
Total	11,22,09,070	24,90,763	2.12

## Similarities of COVID-19 With Previous Coronavirus-Caused Epidemics/Pandemics (SARS and MERS)

In just two decades of 21^st^ century, the world has witnessed the emergence of three novel coronavirus outbreaks. In the year 2002, first coronavirus crossed the species barrier and caused SARS. Later depending upon the clinical symptoms of the disease, the identified causative virus was named as SARS-CoV (Khan et al., 2020). The epicenter for the SARS-CoV outbreak was the Guangdong province of southern China but due to air travel, it reached the other 19 countries of Southeast Asia, South Africa, North America, and Europe. Over time, it infected 8,605 individuals and caused 774 deaths (CFR = 9.5%) worldwide (Peeri et al., 2020). SARS-CoV was transmitted majorly from one person to another *via* coughing, sneezing, shaking hands, or by contact with the contaminated surfaces (De Groot et al., 2013). The virus utilized the angiotensin-converting enzyme-2 (ACE-2) for entering into the host. Notably, health authorities were able to manage the disease through conventional approaches, like isolation, quarantine, and social distancing with infected/suspected/frontline healthcare workers and eradicate it from human population without any pharmaceutical interventions when the case numbers were small (Zhang et al., 2020b). The last case of SARS-CoV was reported in September 2003 (Bell et al., 2004).

In the year 2012, another novel virus of the family *Coronaviridae* caused *Middle East respiratory syndrome* (MERS) and the causative virus was named as MERS-CoV (De Groot et al., 2013). The epicenter of MERS-CoV was the Arabic peninsula but like SARS, it reached 27 countries across the globe *via* air travel and infected 2,494 individuals worldwide. MERS-CoV caused the deaths of 858 individuals (CFR = 34.4%), but unlike SARS, it was still endemic in the Arabian Peninsula (WHO, 2020a).

SARS-CoV-2 is the third virus of *Coronaviridae* family that has crossed the species barrier and infected humans. Being a member of *Coronaviridae*, it has many similarities with the other two viruses that crossed the species barrier and infected humans (**Table 2**). The knowledge gained during the earlier outbreaks of its ancestors played a critical role in the prevention and control of SARS-CoV-2 infection worldwide.

**Table 2:** Comparative analysis of SARS, MERS, and COVID-19.

Parameters	SARS-2002	MERS-2012	COVID-2019	References
Outbreak year	2002	2012	2019	(Liu et al., 2020b; WHO, 2020a; Yuen et al., 2020)
Outbreak epicenter	Guangdong, South China	African peninsula	Wuhan Central China	
Causative agent	SARS-CoV	MERS-CoV	SARS-CoV-2	
Genome type	(+) ssRNA	(+) ssRNA	(+) ssRNA	
Genome size	29.7 kb	30.1 k	29.9 kb	
Sequence homology	80%	50%	100%	(Belouzard et al., 2012; Jaimes et al., 2020)
Viral Receptor	ACE-2	DDP-4	ACE-2	
Reservoir host	Civet cats and bats	Dromedary camel and bats	Bats	
Disease type	Zoonotic	Zoonotic	Zoonotic	(Jia et al., 2005)
Incubation period	6.4 Days (Range 2–7 Days)	5 Days (Range 2–14 Days)	1–14 Days	(Zhu et al., 2020b)
R_0_ value	1.7–1.9	<1	1.4–3.58	(Qu et al., 2020)
No. of infected persons	8,605	2,494	~98.79 million (as of January26, 2020)	
No. of deaths (CFR %)	774 (9.5%)	858 (34.4%)	~2.12 million 2.02%	
Total no. of effected countries	19	27	215	

## Transmission of SARS-CoV-2

COVID-19, being a zoonotic disease, is caused by pathogens which usually infect animals but can infect humans in specific conditions (Mackenzie and Smith, 2020). Therefore, identification of the animal hosts/reservoirs of the SARS-CoV-2 and its transmission from animals-to-human and human-to-human is critically important in order to control the diseases and hence efforts are ongoing for the same (Ye et al., 2020).

### Animal Reservoirs and Animal-to-Human Transmission

The exact natural reservoir(s) and intermediate host(s) of SARS-CoV-2 are unknown yet. However, zoonotic animals such as bats, Himalayan palm civets (*Paguma larvata*), and raccoon dogs (*Nyctereutus procyonoides*) that are sold in the exotic animal market are being considered as its potential hosts. SARS-CoV-2 virus genome shares 96.2% genome sequence identity with Bat-CoV (RaTG13) and 80% with SARS-CoV indicating that Bat-CoV, SARS-CoV, and SARS CoV-2 might share the same ancestor (Wang et al., 2006). Many residues of spike protein of SARS-CoV-2 have also been observed to be conserved across many coronaviruses species indicating the possibility of having other animals (such as turtles, pangolin, minks, and snakes) that have served as host for other coronaviruses as its alternative intermediate hosts (Wang et al., 2006). At the same time, the surface glycoproteins have also shown remarkable variations indicating the potential presence of a receptor-switching mechanism (spike modularity) that might help the virus to bind and infect various mammalian host species. Recently, SARS-CoV-2 has been reported to infect the ferrets and the virus was observed to shed in saliva, nasal fluid washes, urine, and feces till the 8 days post-infection (Kim et al., 2020b). The direct (when infectious particles are directly transferred to the receiver) and indirect (when infectious particles are transferred into the microenvironment/surfaces and receivers enters into this microenvironment and gets infected) contact of naive ferrets with the positive ferrets made naive ferrets shed positive for viral RNA (Kim et al., 2020b). Therefore, ferrets are currently being used as an infection and transmission animal model for development of therapeutics and vaccines. Moreover, Syrian hamsters have also been demonstrated recently to be a good animal model for SARS-CoV-2 infection (Imai et al., 2020). Direct or indirect contact with infected host animals or its consumption is considered as one among the main routes of SARS-CoV-2 transmission (Zheng, 2020).

### Human-to-Human Transmission

Human-to-human transmission is primarily observed between family members and close relatives/friends who may have come in contact with patients or asymptomatic carriers (Gao et al., 2020b). The virus is typically transferred by the patient/carrier through coughing and sneezing and then the droplet are inhaled or contact-transmitted through oral, nasal, or conjunctiva contact by the receiving person present within the 1 meter distance from the infected person (Van Doremalen et al., 2020a). Such transmission can be minimized by maintaining physical distancing. The patient/carrier may also infect the surfaces by direct (contact) or indirect (coughing, sneezing, etc.) routes which could then initiate inoculation in the receiving person once get in contact of such surfaces and transfers the infection to the mouth, nose, or eyes; such infections could be minimized by sanitizing surfaces before touching (WHO, 2020c). Several investigations suggest that SARS-CoV and MERS-CoV can survive on dry surfaces for further transmission (Scagliarini and Alberti, 2020). MERS-CoV could survive at a low temperature and low humidity conditions and infect even after 48 h of surface contamination (Van Doremalen et al., 2013). Similarly, the SARS-CoV-2 can also survive on dry surfaces for longer period of time and may cause infections; and hence may require more effective prevention and control strategies. Recently, SARS-CoV-2 was reported to be stable and viable on various surfaces (plastic, stainless steel, glass, ceramics, wood, latex gloves, and surgical mask) for 7 days and the titer was reducing slowly over time (Van Doremalen et al., 2020a). For example, it’s TCID50/ml decreased from 105.83 to 102.06 after 7-days on plastic (~3.8 log_10_ reduction from the original inoculums). In China alone, out of total infected patients, 72.3% got infected by coming in contact with the infected residents and the infected visitors from the Wuhan epicenter (Guan et al., 2020). Therefore, the frontline health workers and coworkers are at the highest risk of getting infected and may become the most potential source of transmitting the SARS-CoV-2 to other humans like family members, co-workers, etc. (Ali et al., 2020).

A few other transmission routes have also been reported in sporadic studies; these includes airborne transmission, feco-oral transmission, transmission through pregnancy and breastfeeding, sexual transmission, and transmission through tears and conjunctival secretions.

#### Airborne Transmission

Airborne transmission, unlike droplet transmission, occurs when the microbes are present in the air for long periods of time and infects others over distances >1 meter (Kumar et al., 2020a). The predecessor of SARS-CoV-2, SARS-CoV-1 was known to spread through air (Morawska and Cao, 2020). A number of COVID-19 cases were also found in the areas outside Wuhan in China, such as Hunan and Tianjin suggesting the possibilities of non-contact transmission of SARS-CoV-2 (Wang and Teunis, 2020). Earlier during spread of SARS-CoV-2 in Toronto, novel air sampling and surface swabbing was done in rooms occupied by COVID-19 patient to investigate environmental contamination. In the said investigation, two-air samples were found positive for SARS-CoV-2 viral RNA indicating the presence of virus in air (Chia et al., 2020). And another study has also shown that SARS-CoV-2 can have a half-life of 1.1 h in aerosols (21–23°C temperature and 65% relative humidity) and a survival of 3 h in the air (Morris et al., 2020). These data confirmed the viral aerosol generation by a COVID-19 patient establishing the airborne transmission of SARS-CoV-2 and indicating the need of adopting adequate respiratory protection and surface hygiene practices (Cheng et al., 2020).

#### Feco-Oral Transmission

Recently, 39 out of total 73 confirmed SARS-CoV-2 positive patients for the presence of viral RNA in respiratory samples were found positive for the presence of viral RNA in stool sample indicating the possible feco-oral transmission of virus (Amirian, 2020). Interestingly, 23% of 39 patients, who were found positive for viral RNA in stool, also remained positive for viral RNA in stool even when the respiratory samples became negative later on indicating long term shedding of viral RNA in stool (Amirian, 2020). Even after a negative nasopharyngeal swab, viral shedding has been reported in feces up to 33 days after the appearance of symptoms in COVID-19 patients and this can continue up to 47 days (Wu et al., 2020c). Additionally, in a recent study, researchers isolated the high viral load from cultured stool samples and characterized the live virus in culture (Prasad et al., 2020). The ACE-2 protein is profusely found in the glandular cells of rectal epithelia (Kumar et al., 2020a). And SARS-CoV-2 viral RNA and intracellular staining of viral nucleo-capsid protein has been reported in the rectal epithelia confirming that the virus can infect such epithelial cells as well (Xiao et al., 2020). These observations suggest that GI tract could serve as a site of infection and enhance transmission of SARS-CoV-2 (Amirian, 2020; Patel et al., 2020; Xiao et al., 2020). However, it is still not clear if the presence of viral RNA in GI tract is due to consumption of virus-contaminated food or due to the leakage of viral RNA in GI tract. But, it can be considered that if SARS-CoV-2 re-emerges in the future, water contaminated with the fecal waste of infected individuals could potentially also be a vehicle for transmission along with many others (Ding and Liang, 2020).

#### Transmission During Pregnancy and Breastfeeding

Expression of ACE-2 receptors in the reproductive organs such as granulosa, stroma cells, as well as immature oocytes in rat ovaries and in human uterus, vagina, and placenta biologically make it plausible that a pregnant woman may be more vulnerable to SARS-CoV-2 infection and/or serve as carrier (Jing et al., 2020). Recently, there were two cases of neonatal SARS-CoV-2 infection diagnosed at 36 h and 17 days post-deliveries respectively, creating a history of two confirmed cases of SARS-CoV-2 infection (Wang et al., 2020c). Later, a premature newborn from an asymptomatic infected mother was reported RT-PCR positive for SARS-CoV-2 using nasopharyngeal swab samples collected after 24 h of life (Piersigilli et al., 2020). However, it remained unknown if these babies got the SARS-CoV-2 infection before, during, or after birth at hospital site. Recently, there have been reports of SARS-CoV-2 nucleic acid detection in placenta also (Ferraiolo et al., 2020). Moreover, one of the three-breast milk samples, collected from SARS-CoV-2 infected women during different stages of pregnancy, have been found positive for viral nucleic acid testing (Chen et al., 2020a). In another study conducted over 64 pregnant women who delivered, two newborns were found to be SARS-CoV-2 positive by RT-PCR (Shen et al., 2020). Besides, elevated levels of SARS-CoV-2 IgG and IgM have been reported in a 2-hour-old neonate delivered by caesarean (Dong et al., 2020). Contrarily, studies also suggest that there was no evidence of vertical transmission in all the neonates born to 14 pregnant women infected with SARS-CoV-2 (Gao et al., 2020a). Thus, further investigations are needed to understand and establish the potential link between pregnancy, female reproductive organs, breast milk, and the potential risk for viral infection to the mother and the babies.

#### Transmission Through Sexual Contact

Although SARS-CoV-2 has not yet been reported to be sexually transmitted, the possibility of its transmission *via* sexual contact can be logically hypothesized. ACE-2 expression is already reported on the mucosa of oral cavity, rectal, vaginal, and ovarian epithelial cells and in male reproductive organs (high in sperm cells) (Candotto et al., 2017). Thus, certain sexual practices could cause additional ways for SARS-CoV-2 transmission, both directly (e.g. through seminal/oral-anal/vaginal contacts) or indirectly (*i.e. via* exposure of the rectal mucosa or vaginal epithelium to the saliva for lubrication during anal sex) (Gupta et al., 2006). Recently SARS-CoV-2 was reported in the semen samples of six patients, including two subjects who were recovering from the clinical disease (Shen et al., 2020). However, detailed evidences to support the COVID-19 transmission *via* semen or vaginal fluids are scarce and hence demands further research to validate the observations of its sexual transmission.

#### Transmission Through Tears and Conjunctival Secretions

SARS-CoV-2 RNA has been found in tears and conjunctival secretions collected from SARS-CoV-2 infected patients which was confirmed through a study reporting SARS-CoV-2 viral replication in Vero-E6 cells inoculated with ocular sample collected from a SARS-CoV-2 positive patient from Italy and having bilateral conjunctivitis (Chen et al., 2020b). However it is not clear if the virus can replicate in the conjunctiva (Kumar et al., 2020b). Therefore, it is highly possible that SARS-CoV-2 can transmit through the ocular surface. Recently a few ophthalmologists who wore an N95 mask but nothing to protect eyes have been found to be infected with SARS-CoV-2 while dealing with COVID-19 patients (Lu et al., 2020a). However, more targeted efforts are required to establish the facts.

## Molecular Mechanism of SARS-CoV-2 Infection

The ACE-2 serves as a receptor for the SARS-CoV-2, like SARS-CoV. The interaction between homotrimers of the spike protein of the SARS-CoV-2 with ACE-2 facilitates the viral entry into the host and its establishment for pathogenesis. ACE-2 binds to the spike protein of SARS-CoV-2 with higher affinity compared to SARS-CoV indicating its suitability for the more efficient spread and hence infectious nature (Wu et al., 2020a).

The spike glycoprotein consists of two functional subunits, S_1_ that binds with the host ACE-2 receptor and S_2_ that mediates the fusion of viral and host cellular membrane (Belouzard et al., 2012). For entry of many coronaviruses into the host, cells require priming of the spike protein by the host proteases which cleave the spike protein at the boundary between the S_1_ and S_2_ subunits at the S_2_` cleavage site and allows the fusion of viral and cellular membrane (Belouzard et al., 2012). Interestingly, SARS-CoV-2 has a unique furin cleavage site, present at the S1/S2 interface of the spike, which is absent in SARS-CoV and other SARS-related coronaviruses (Jaimes et al., 2020). The protease that mediates this cleavage have been identified as TMPRSS2 (Jaimes et al., 2020). Recently, abrogation of this cleavage has been shown to affect the viral entry into the Vero-E6 cells indicating the potential role of furin site infusion, tropism, and pathogenicity (Hoffmann et al., 2020). Therefore, anti-spike antibodies isolated from infected but recovered COVID-19 patients and inhibitors of TMPRSS2 proteases (such as camostae mesylate) are being considered as potential therapeutics to treat the diseases and are under clinical trial.

**Figure 3:**
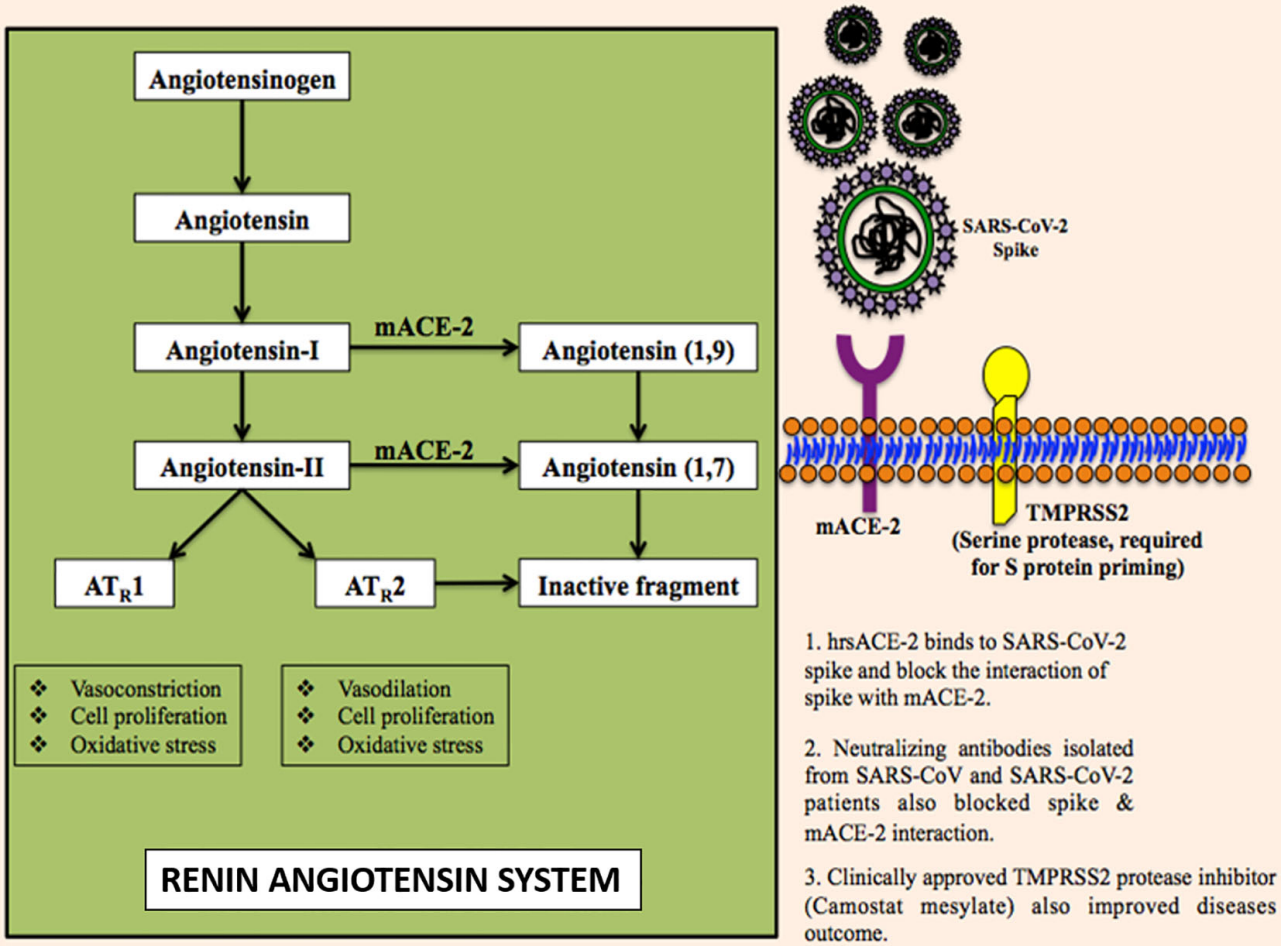
The molecular mechanism of SARS-CoV-2 infection.

The ACE-2 is found in a wide range of cells and tissues. It is prominently expressed in the alveolar epithelial type II cells in lungs, heart, kidney, retina, and uteroplacental tissue (Jia et al., 2005). It is the part of the renin-angiotensin-aldosterone system (RAAS) which is well known for regulating blood pressure, electrolytic homeostasis, and heart remodelation (Muñoz-Durango et al., 2016). Abnormal activation of RAAS has been associated with cardiovascular and renal diseases such as hypertension, myocardial infarction, and heart failure (Ma et al., 2010). RAAS comprises a cascade of vasoactive peptides that includes prorenin, renin, angiotensinogen, ACE-I, ACE-II, angiotensin-I (Ang-I), and Ang-II (**Figure 3**) (Sparks et al., 2014). Ang-II is a major effector molecule in RAAS that mediates its function by binding to angiotensin II receptors, AT_1_ and AT_2_ (Benigni et al., 2010). Both, AT_1_ and AT_2_ are cell surface receptors that work antagonistically; AT_1_ mediates vasoconstriction and increases the blood pressure, while AT_2_ mediates vasodilation and decreases the blood pressure (Kawai et al., 2017). Once engaged with spike protein, ACE-2 becomes unable to convert Ang-I to Ang1–9 and Ang-II to Ang1–7 resulting in the accumulation of Ang-II that ultimately causes reduced cardiac contractility and cardiac hypertrophy (Gheblawi et al., 2020). The administration of candesartan, an AT_1_ receptor blocker, has been shown to attenuate the hypertrophic response (Villapol et al., 2012). Therefore, blocking the interaction between spike protein and ACE-2 receptor is among the most promising options to treat the COVID-19 disease (Verdecchia et al., 2020). Recently some of the researchers have suggested the potential use of human recombinant soluble ACE-2 (hrsACE-2) and repurposing angiotensin receptor blockers (such as losartan, valsartan, and telmsartan) that are generally used to control the blood pressure, treating heart failure and preventing renal failure, to control the SARS-CoV-2 infections (Furuhashi et al., 2020).

## The Emergence of Dominant D614G Spike Variant of SARS-CoV-2

The SARS-CoV-2 has evolved and accumulated several pathogenicity and/or immunologically relevant mutations in its genome, like other members of *Coronaviridae*, compared to parent reference strain decoded from Wuhan (Guzzi et al., 2020). According to the Global Initiative on Sharing All Influenza Data (GISAID) genome sequencing data of 3,470 samples, a total of eight major clades (clade GR, GH, G, S, V, O, L) has been identified. D614Gspike variant, which consists of glycine (G) in place of aspartate (D) at the sequence position of 614 in the spike protein due to A-to-G nucleotide mutation at position 23,403 in the genome, is categorized in clade G. It is the most prominent strain (~75%) worldwide compared to its parental strain (clade L) which accounts for ~7% only (Isabel et al., 2020). Historic evidences of point mutation in MERS-CoV and SARS-CoV-1 have demonstrated such mutations beneficial to the pathogenicity and survival of the pathogen and hence are being investigated for SARS-CoV-2 as well (Tang et al., 2014). Recently, D614G has been associated with three other mutations: a C-to-T mutation in the 5’ UTR (position 241 relative to the Wuhan reference sequence), a silent C-to-T mutation at position 3,037 and a C-to-T mutation at position 14,408 resulting in an amino acid change in RNA-dependent RNA polymerase (RdRp P323L) (Hou et al., 2020). Genome-based mutation and single-nucleotide polymorphisms (SNPs) studies have also reported overall 205,482 amino acid-changing SNP events, of which, the C>T transition accounted for 55.1%, A>G for 14.8%, and G>T for 12.0% worldwide (Mercatelli and Giorgi, 2020). Structural analysis of G614 variant was reported to prevent the side-chain hydrogen bonding between the promoter of S1 and S2 units and thus increasing the side-chain flexibility and potentially facilitating infectivity of the virion (Walls et al., 2020). Recently, hamsters infected with G614 variant were reported to produce high infection titers of the virus in the upper respiratory tract suggesting enhanced suitability of the G614 spike variant for viral transmission (Plante et al., 2020b). Besides, G614 variant is shown to exhibit increased efficiency of cellular entry and viral replication compared to D614 variant across a broad range of human cell types, including cells from lung, liver, and colon (Daniloski et al., 2020). Furthermore, in the decay of infectivity experiment, G614 spike variant was observed to retain higher infectivity as compared to original D614 spike variant (parent strain) at different temperatures (33, 37, and 42°C) indicating t10he potential role of D614G mutation in stability of SARS-CoV-2 (Plante et al., 2020a).

Although G614 spike variant has been spreading faster and dominated the world, it is still uncertain whether this will have a clinical impact on COVID-19 disease progression. More studies need to be done to find the association between D614G spike variant and disease severity. These findings will be extremely useful in designing efficacy of the measures that have been taken on a regional basis to limit SARS-CoV-2 spreading.

## COVID-19 Diagnostics

Early and accurate diagnosis of COVID-19 patients enables the targeted implementation of quarantine, community containment, and supportive treatment approaches to control and cure the disease and therefore, it is an important tool for better management of the diseases. Although multiple diagnostic assay platforms have been developed and commercialized to date, currently molecular-diagnostics based approaches have been the technique of choice for confirming the infection (**Table 3**). These commonly utilized assay platforms can be divided into two broad categories.

**Table 3:** List of a few currently available diagnostic assays for the detection of COVID-19 across the globe.

Method/Test name/ Manufacturer/ Organization name	Sample collection	Target/Gene	Sensitivity	Specificity	Processing time	Cost	Site of use	References
1) RT-PCR	(i) Nasopharyngeal swab (ii) Sputum (iii) Stool (iv) Tracheal aspirate (iv) Brachial aspirate (v) BAL (vi) BAL (vii) Human EDTA plasma (viii) Oropharyngeal swabs	(i) ORF1ab/RdRp (RNA dependent RNA polymerase) (ii) Envelope protein (E) (iii) Nucleocapsid (N) (iv) Spike (S) protein	69–100 %	77–90 %	1–4 h	H	T	(Tahamtan and Ardebili, 2020)
1.1) Xpert® Xpress SARS-CoV-2	(i) Nasopharyngeal (ii) Oropharyngeal (iii) Nasal wash/aspirate	E & N2	95	NA	2 h	H	T	(Loeffelholz et al., 2020; Wolters et al., 2020)
1.2) LabGun COVID-19 Assay plus	(i) Nasopharyngeal (ii) Oropharyngeal (iii)Nasal aspirate (iv) Sputum	E & RdRp	95	1.8 × 103 NDU/m	3–4 h	H	T	(LabGenomics, 2020)
1.3) Pathodetect Coronavirus (COVID-19) Qualitative PCR Kit	(i) BAL nasopharyngeal (ii) Sputum (iii) Serum (iv) Tissue	E & RdRp	100	100	3–30 h	H	T	(mylabdiscovery, 2020)
1.4) STANDARD M nCoV Real-Time Detection kit	(i) Nasopharyngeal (ii) Oropharyngeal (iii) Midturbinate nasal swab (iv) Sputum specimens	E & RdRp	95	NA	1.5–2 h	H	T	(SDBiosensor, 2020)
1.5) QuantiTect Virus +Rox Vial kit (QIAGEN, Hilden, Germany)	(i) Nasopharyngeal swabs (NPS) (ii) Sputum	E & RdRp	0.97	0.92	2–3 h	H	T	(Böhmer et al., 2020)
1.6) Real-Time Fluorescent RT-PCR Kit for Detecting SARS-CoV-2 (BGIGenomics Co. Lt)	(i) Oropharyngeal swabs (ii) BALF	ORF1ab	69.1–89.1%	77.0–97.0%	1–2 h	H	T	(Afzal, 2020)
2) LAMP	(i) Nasopharyngeal swab (ii) Sputum (iii) Stool (iv) Respiratory secretions (v) Plasma (vi) Serum	(i) ORF1ab/N (ii) RNA of SARS CoV-2 (iii) IgG/IgM	52–100%	43–100%	5–35 min	L/M	T	(El-Tholoth et al., 2020)
2.1) Loopamp®2019-SARS-CoV-2 Detection Reagent Kit	Nasopharyngeal swab	RNA of a SARS-CoV-2	100%	97.60%	35 min	M	T	(Kitagawa et al., 2020)
2.2) Abbott ID NOW COVID-19 test (Abbott 125Diagnostics, Lake Forest, IL)	(i) Plasma (ii) Serum	IgM/IgG	52–97%	43–99.62%	5–13 min	L	T	(Shah, July 13, 2020)
2.3) COVID-19 Rapid Isothermal PCR Kit	Nasopharyngeal swabs	Nucleocapsid (N) protein	25 viral RNA copies / µl	NA	30 min	M	T	(Raybiotech, 2020)
3) Immunoassays	(i) Serum (ii) Plasma (iii) Whole Blood	Antibody detection	58–81% (IgM) 53–98% (IgG) 80–99% (IgM+IgG)	83.1–100%	10 min–3 h	M	T	(Jääskeläinen et al., 2020)
3.1) Maglumi™2019-n-Cov IgG and IgM	(i) Serum (ii) Plasma (iii) Whole Blood	IgM and IgG against viral recombinant antigen	IgM (58.7%) 53.2% (IgG) 64.30% (IgA/IgG)	94.9–100 % (IgM) 94.9–100% (IgG) 94.9–100 % (IgA/IgG)	30 min	M	T	(Montesinos et al., 2020)
3.2) EuroimmunAnti-SARS-CoV-2 IgG and IgA assay	Serum	IgG and IgA against S1 structural protein	83.6% (IgA) 61.7% (IgG) 84.40% (IgA+IgG)	83.1% (IgA) 98.6% (IgG) 87.50% (IgA+IgG)	3 h	M	T	(Nicol et al., 2020)
3.3) Atellica IM SARS-CoV-2 Total (COV2T), Chemiluminescent microparticle immunoassay, Siemens Healthcare	(i) Serum (ii) Plasma	Total antibody against RBD of S1 protein	14 days post-symptom onset:100%	14 days post-symptom onset:99.8%	~10 min	M	T	(Smithgall et al., 2020)
3.4) Abbott ARCHITECT i2000SR	(i) Serum (ii) Plasma	IgG against nucleocapsida protein (NCP)	97–100%	100%	2–3 h	M	T	(Nicol et al., 2020)
3.5) Covid Kavach Elisa, Indian Council for Medical Research (ICMR)	Blood	IgG-based ELISA	92.37%	97.90%	2–3 h	M	T	(Sapkal et al., 2020)
3.6) LIAISON® SARS-CoV-2 IgM & IgG, DiaSorin assays	Serum	IgM and IgG against S1/S2 protein	≤7 days: 69.5% (60.2–77.5%) 8–14 days: 91.5% (80.1–96.6%) 15–30 days: 98.3% (93.9–99.5%)	99.20%	2–3 h	M	T	(Bonelli et al., 2020)
3.7) Roche’s SARS-CoV-2 antibody test, Roche Diagnostics	Blood	IgM and IgG	87.0%	100%	18 min	M	T	(Burki, 2020)
4) Lateral Flow	(i) Blood (ii) Serum (iii) Plasma	Antibody detection	48–84%	31–100%	5–20 min	L/M	P	(Wu et al., 2020b)
4.1) COVID-19 IgG/IgM Rapid Test Cassette (Premier Biotech, Minneapolis, MN)	(i) Whole blood (ii) Serum (iii) Plasma	IgG/IgM	82.80%	99.50%	12–20 min	M	P	(Dobaño et al., 2020)
4.2) STANDARD Q COVID-11619 IgM/IgG Duo Test kits (SD Biosensor, Gyeonggi-do, Korea)	(i) Whole blood (ii) Serum (iii) Plasma	IgM/IgG	11% in early infection and up to 100% beyond 14 days of infection	43–85.7%	15 min	L	P	(PIH, 2020)
4.3) COVID-19 Ag Respi Strip	Oropharyngeal swab in VTM	SARS-CoV-2 antigen	30.20%	100%	15 min	L	P	(Scohy et al., 2020)
4.4) BIOCARD Pro COVID-19 Rapid Ag test kit	Human Nasopharyngeal Swab	Covid 19 Antigen	83%	98%	5–7 min	L	P	(Trivitron, 2020)
4.5) Feluda paper strip test	Nasopharyngeal	SARS-CoV-2 antigen	96%	98%	45 min	L	P	(ICMR, 2020a)

#H, High; M, Medium; L, Low; P, Primary Care; T, Tertiary Care.

### Nucleic Acid-Based Diagnostics

There are two nucleic acid-based diagnostic platforms, real-time Reverse Transcription-Polymerase Chain Reaction (RT-PCR) and Loop-mediated isothermal amplification (LAMP). Of these, RT-PCR is considered as a gold standard. Both the tests amplify and detect the presence or absence of SARS-CoV-2 specific gene(s) in the RNA isolated from Nasopharyngeal (NP)/Oropharyngeal swab or sputum of the subject (Wang et al., 2020e). Notably, the collection of these clinical specimens also requires skilled manpower risking them for viral exposure and is a time-consuming process.

#### RT-PCR

The majority of currently available RT-PCR based SARS-CoV-2 diagnostics are designed to identify the presence of viral genomic RNA translating coronavirus structural proteins, i.e. ORF1ab/RdRp (RNA dependent RNA polymerase), envelope (E), nucleocapsid (N), and/or spike (S) proteins (Kim et al., 2020a). RT-PCR can detect active symptomatic/asymptomatic infections with high sensitivity and specificity (Yang and Rothman, 2004). It can analyze multiple samples simultaneously and so offers scalability. RT-PCR being laborious, time-consuming, requiring high-end equipment and trained manpower, it’s been of limited use in the resource-constrained settings. However, a recent study has shown the use of direct swab sample which will further simplify the test by eliminating the RNA isolation step (Kiran et al., 2020).

#### LAMP

LAMP-based tests for SARS-CoV-2 detection may serve as the future-technique for the purpose. LAMP amplifies the target at a constant single-step temperature of 60–65°C, in contrast to RT-PCR which utilizes a series of temperatures and hence LAMP requires a basic level of equipment and laboratory set-up and is easy-to-perform (Udugama et al., 2020). The amount of DNA produced after amplification in LAMP is also much higher (~10–100 fold) than the RT-PCR primarily due to single-temperature amplification offering the possibility for visual detection (turbidity) of the amplification (Fakruddin et al., 2013). LAMP assay is comparatively quick and can produce the results within 1–3 h. A few RT-LAMP based assays have already been commercialized and are being used for testing purposes. The elimination of RNA extraction step may also be optimized for the RT-LAMP for further reducing the cost and turn-around-time of the assay.

### Serological and Immunological Assays

Nucleic acid-based methods can diagnose active infection but are of limited use for monitoring the disease progression and identification of past infection and immunity development (Niemz et al., 2011). Therefore, another subset of tests for qualitative or quantitative assessment of immune response to the virus in patients by detecting the presence of IgM (early-stage and/or primary infection) and IgG antibodies (current-stage and/or prior infection) are being developed (Jacofsky et al., 2020). Currently, S protein (S1 and S2 domains and RBD) and/or N protein are the most common antigens being used in enzyme-linked immunosorbent assay (ELISA) format for quantitative detection and lateral flow rapid Assay (LFA) for qualitative format (Sheikhzadeh et al., 2020). Both the formats have their advantages and disadvantage concerning the requirement of sample preparation, equipment, and trained manpower along with turn-around-time, cost, and field-deploy ability. ELISA offers the possibility to analyze multiple samples simultaneously with high sensitivity using a relatively simple process, though; it is laborious and requires equipment and trained manpower (Roshanravan et al., 2020). Whereas, Lateral Flow Assays (LFAs) are the simplest, fit for field monitoring, rapid, user-friendly, and cost-effective. However, it gives qualitative results that limit their use for serological studies. Many of the ELISA and LFA has been approved and commercialized under Emergency Use Authorization (EUA) by the FDA without due detailed validation (Hahn, 2020).

### Others

The work is ongoing to develop and commercialize LFA-based methods for viral-antigen detection using non-invasive (saliva) or minimally invasive (nasal swab and finger-prick whole-blood) clinical specimens (Corstjens et al., 2012). For this, several laboratories have a special focus on developing diagnostic grade binders (antibody, nucleic acid, aptamers, etc.) (Wang et al., 2020e).

Recently, CRISPR-Cas9 based technology for diagnosis of SARS-CoV-2 has also been approved for commercialization. Along with others, TataMD’s CHECK will be commercializing the CRISPR-Cas9 based diagnostic assay in India (ICMR, 2020b).

Moreover, imaging techniques (X-Ray and CT-Scan) are being utilized to review the disease progression or confirming the diagnosis of suspected patients who are at high risk of COVID infection clinically but show negative (false) RT-PCR results.

## COVID-19 Therapeutics

Since SARS-CoV-2 is highly infectious in nature, effective treatment is an urgent global need. Researchers have been focusing on developing new anti-viral molecules as well as exploring the repositioning of FDA-approved molecules to inhibit viral entry or its replication to control/treat the infection (Duan et al., 2020). A few molecules that have shown promising results in early evaluation have been listed in **Table 4**. These include:

**Table 4:** Representative list of currently used pharmaceutical interventions for treatment of COVID-19 across the globe.

Drug name	Commercial name	Class	Approved for	Recommended doses	Clinical trial NCT No.	Status of clinical trial (Dec 2020)
Molnupiravir	Molnupiravir	Antiviral	Influenza virus	Twice—5 mg kg−1 body weight	NCT04575584	Phase 2/3
Remdesivir	Remdesivir	Antiviral	Ebola virus	Day1: 200 mg Day 2–10: 100 mg (for serious ill patients) Day 2–4: 100 mg (for mild patients)	NCT04257656	Phase 3
Lopinavir/Ritonavir	Kaletra	Antiviral	Influenza virus	Day 1–14: 2×400/100 mg orally	NCT04321174	Phase 3
Chloroquine and Hydroxychloroquine	Aralen and Plaquenil	Antimalarial	Malaria	Day 1: 1 gm Day 2–7: 500 mg	NCT04303507	Not Applicable
Favipiravir	Avigan	Antiviral	Influenza virus	Day 1: 1,800 mg, BID Day 2–14: 600 mg TID	NCT04336904	Phase 3
Umifenovir	Arbidol	Antiviral	Influenza virus	Day 1–14/20: 2 tablets/time, 3 times/day	NCT04260594	Phase 4
Ivermectin	Soolantra	Antiparasitic	Oncocerciasis	Day 1–7: 0.2 mg/kg (single dose at once = 2 tablets of 6 mg/weekly	NCT04343092	Phase 1
Tocilizumab	Roactemra	Monoclonal antibody	IL-6	Once: 8 mg/kg bodyweight, max. Single dose 800 mg) (active ingredient: TCZ) intravenously in 100 ml NaCl 0.9% Infusion time: 60 min	NCT04335071	Phase 2
Sarilumab	Kevzara	Monoclonal antibody	IL-6	Solution for injection administrated intravenously	NCT04327388	Phase 3
Camostat Mesylate	Camostat	Antiviral	Esophagitis	Day 1–7: 3×3,200mg taken orally	NCT04353284	Phase 2
Anakinra	Kineret	Immunosuppressor	IL-1α and IL-1β	Day 1–28: subcutaneous injection of 100 mg	NCT04330638	Phase 3
Ravulizumab	Ultomiris	Monoclonal antibody	Paroxysmal Nocturnal Hemoglobinuria (PNH)	Weight based doses given at Day 1, 5,10, and 15	NCT04369469	Phase 3
Aviptadil	Aviptadil	Synthetic peptide (vasodilator)	ARDS	50–150 pmol/kg/h over 12 h	NCT04311697	Phase 2
Tradipitant	Tradipitant	Neurokinin-1 receptor antagonist	Gastroparesis, motion sickness, and atopic dermatitis	2×85 mg orally	NCT04326426	Phase 3
Otilimab	Otilimab	Monoclonal antibody	Rheumatoid arthritis	Administered once *via* IV route	NCT04376684	Phase 2
Nafamostat mesylate	Nafamostat Mesylate	Inhibitor	Cystic Fibrosis	Administered intravenously as a continuous infusion	NCT04352400	Phase 2/3
Eculizumab	Soliris	Monoclonal antibody	Complement C5	Day 1–7: 900 mg IV after ceftriaxone IV	NCT04288713	Not Applicable
Baricitinib	Breath	JAK inhibitor	Rheumatoid arthritis	4 mg/day for 7 days	NCT04399798	Phase 2
Enzalutamide	Covisenza	Antiandrogen	Prostate cancer	5 days with 4×40 mg enzalutamide tablets orally once daily	NCT04475601	Phase 2
Zotatifin	Propel	Signaling molecules	Solid Tumor Malignancies	0.035 mg/kg zotatifin	NCT04632381	Phase 1
Colchicine	Colcovid19	Anti-gout agent	Gout	0.5 mg	NCT04539873	Phase 3
Nitazoxanide	Nitazoxanide	Antiprotozoal agent	Diarrhea	500 mg, orally	NCT04382846	Phase 3
Losartan	Losartan	Angiotensin II receptor antagonists	Hypertension	50 mg daily, oral	NCT04312009	Phase 2
Dipyridamole	Dicer	Nucleoside transport inhibitor	Thromboembolic complications	100 milligram (mg)	NCT04391179	Phase 2

*Not Applicable is used to describe trials without FDA-defined phases, including trials of devices or behavioral interventions.

### Viral Entry Inhibitors

The viral entry inhibitor molecules stop the entry of SARS-CoV-2 into the host cell and hence its pathogenesis. Several molecules have been explored for the purpose and a few potential candidates as listed below were identified.

#### Chloroquine and Hydroxychloroquine

Chloroquine is the most commonly used drug to treat malaria that is caused by several species of *Plasmodium* (Van Doremalen et al., 2020b). Chloroquine alters glycosylation of ACE-2 decreasing the affinity of ACE-2 with spike protein and ultimately reduces the viral entry into the cell (Vincent et al., 2005). Chloroquine has also been reported to increase the endosomal pH required for viral fusion and hence block viral entry into the host (Wang et al., 2020a). Chloroquine along with hydroxychloroquine also inhibits the Toll-like receptor (TLR) pathway that regulates pro-inflammatory cytokine signaling and hence may provide symptomatic relief. Chloroquine 500 mg twice a day for 10 days was recommended to treat the COVID-19 infected patients, however, doses >5 grams caused ventricular dysrhythmias and hypokalemia resulting in high mortality (Yasuda et al., 2008). Recently, 25 out of 31 COVID-19 patients were clinically improved after receiving hydroxychloroquine in comparison to 17 out of 31 in the no-hydroxychloroquine treated COVID-19 patients control group (Cao et al., 2020b). However, one patient was observed to develop rashes and another developed headache following hydroxychloroquine treatment which later got resolved without any intervention (O’Neill and Netea, 2020). In China and France, chloroquine phosphate has also been shown to provide relief against COVID-19 caused pneumonia in sporadic studies and hence may be further explored through randomized trials (Oliver, 2020). Moreover, azithromycin in combination with Chloroquine/hydroxychloroquine has also been shown to have beneficial effects against SARs-CoV-2 infection (Singh et al., 2020). However, the studies to date have shown the mix-effect (beneficial or no-effect) of Chloroquine/hydroxychloroquine on COVID-19 patients (NCT04333654, NCT04329923, NCT04321993). Therefore, further clinical studies are required to conclude the observations and provide guidance to clinicians and policymakers.

#### Recombinant Human Angiotensin-Converting Enzyme-2 (rhACE-2, APN01)

APN01 (Apeiron Biologics) originally developed for Acute Respiratory Distress Syndrome (ARDS) and already undergone phase II clinical trial is a soluble molecular drug for SARS-CoV-2. The soluble rhACE-2 blocks SARS-CoV-2 entry into the host cells by inhibiting the spike-protein interaction with the host cellular ACE-2 and hence reduces the acute lung injury (Groß et al., 2020). It is also believed that the administration of rhACE-2 can reduce ang-II levels in the serum by directing the substrate far-away from the related enzyme inhibiting the activation of the ACE-2 receptor and thereby retaining the pulmonary vascular integrity and avoiding ARDS (Roshanravan et al., 2020). APN0l has been reported safe with no immunogenicity and cardiovascular side effects in clinical trials (Li et al., 2019). Recently engineered trimeric ACE-2 variant have also been reported to be anti-SARS-CoV-2 and hence helpful for treating COVID-19 patients (Xiao et al., 2021).

#### Leronlimab (PRO-140)

Leronlimab is a humanized IgG4 antibody targeting chemokine receptor 5 (CCR5) found on T-lymphocytes (Chary et al., 2020). CCR5 is a co-receptor that facilitates human immunodeficiency virus (HIV) entry into the host, white blood cells (Wilen et al., 2012). Later, other pathogens (like Dengue or *Staphylococcus aureus*) were also observed to use the CCR5 signaling pathway for their entry or as a virulence factor (Alonzo et al., 2013). At present, leronlimabis under a “fast-track” designation by FDA to treat HIV and metastatic breast carcinoma (Jiao et al., 2019). A Phase 2 clinical trial to evaluate the safety and efficacy of leronlimab (PRO 140) (700 mg/week) in COVID-19 patients with prolonged symptoms is under progress and is estimated to be completed by June 2021 (NCT04678830).

### Viral Replication Inhibitors

RNA viruses utilize the host machinery to make a copy of its RNA genome and synthesize proteins that are required to form new virions, such as capsid and spike proteins. The inhibition of viral RNA replication and protein synthesis is being considered as a valuable step to treat and control SARS-CoV-2 infection. The present inhibitors for the aim include:

#### Nucleotide Analogs

Nucleotide Analogs is a class of anti-cancer and anti-viral chemotherapeutics, which is used to inhibit the synthesis of new viral RNA, restricting the infected cells to become synthesis sites for new virions. Nucleotide analogs hinder the elongation of the viral replicating strand by incorporating itself (base analog) into it and thus the viral RNA polymerase cannot elongate. Some of these drugs include:

##### Molnupiravir

Molnupiravir (EIDD-2801), a pro-drug of N4-hydroxycytidine, is an orally active antiviral drug that was developed to treat influenza. It gets hydrolyzed *in vivo* to acquire its active triphosphate form and then gets incorporated into the viral genome thus leading to mutation and copying errors. Studies have shown EIDD-2801 inhibits replication of human and bat coronaviruses, including SARS-CoV-2, in mice and human airway epithelial cells (Sheahan et al., 2020). After clearing the safety, tolerability, and pharmacokinetics preliminary studies in the healthy subject, this drug was tested for its efficacy in a phase-2 trial in June 2020. And in October 2020, the clinical trial phase-2/3 focusing on the hospitalized COVID-19 patients has been started. A recent study on the treatment of infected ferret with MK-4482/EIDD-2801 has shown significant reduction in SARS-CoV-2 load in the upper respiratory tract with complete suppression of SARS-CoV-2 spread to untreated contact animals (Cox et al., 2020).

##### Remdesivir

Remdesivir, a broad-spectrum antiviral, was originally developed for treating the Ebola virus infections and is considered by far the most-promising against RNA viruses (Tchesnokov et al., 2019). It is a pro-drug metabolized to an adenosine nucleotide analog, which gets integrated into nascent viral RNA and inhibits RNA-dependent RNA polymerase enzyme leading to premature termination of the viral RNA chain and blocking viral genome replication. In previous studies, remdesivir was found effective against SARS-CoV and MERS-CoV and hence evaluated for its potential to treat SARS-CoV-2 infections and shown to inhibit the virus (Cao et al., 2020b). Remdesivir was used for treating the first patient of COVID-19 in the USA on the 7th day after hospitalization and within 24-h of treatment, the patient’s condition was considerably improved without any noticeable side effect (Cao et al., 2020b). The combination of remdesivir with an inflammatory drug, baricitinib, has recently shown to increase its potential to reduce viral infection. However, due to the unavailability of data on remdesivir toxicity, it can be extrapolated from the toxicity reported for other nucleoside analogs to check several parameters which include severe metabolic acidosis, peripheral neuropathy, bone marrow suppression, pancreatitis, and myopathy, most possibly due to mitochondrial dysfunction with several medications in this class (Wang et al., 2020b). A clinical trial (NCT04280705) has now reported that Remdesivir helps in shortening the time to recovery in COVID-19 affected hospitalized adults with an infection in the lower respiratory tract, however another trial (NCT04257656) has concluded no statistically significant clinical benefits thus demanding for further conformational studies.

##### Favipiravir

Favipiravir is also an inhibitor of the RNA-dependent RNA polymerase that structurally resembles the endogenous guanine (Jin et al., 2013). It is already known that the efficacy of viral replication can be hugely reduced through competitive inhibition. Favipiravir, although being approved for influenza treatment, has established less preclinical support to treat COVID-19 as compared to remdesivir (Fosun, 2020). However, COVID-19 patients have been recently recruited to study the potential of favipiravir in combination with IFN-α (ChiCTR2000029600) (Gao et al., 2020c). In March 2020, a clinical trial demonstrated the efficacy of favipiravir with minimal side effects and thereafter favipiravir was permitted as the first anti-SARS-CoV-2 drug to be used in China by the National Medical Products Administration of China (Tu et al., 2020). However, according to a recent study, inclusion of favipiravir at EC_50_ resulted in no additional antiviral benefit to the existing standard treatment (Lou et al., 2021). Hence, further investigations are required to conclude its impact on COVID disease management.

##### Umifenovir

Umifenovir is a broad-spectrum antiviral drug primarily used to treat influenza in Russia and China. It was first licensed in 1993 in Russia (brand name: Arbidol) and 2006 in China (Proskurnina et al., 2020). Various clinical studies have previously reported the potential of umifenovir in reducing the SARS virus reproduction, however, its potential in treating COVID-19 is underexplored (Lian et al., 2020). In the initial study, comparison of clinical characteristics and outcomes among the COVID-19 patients who received or did not receive the umifenovir treatment, the drug was observed ineffective in clearing the SARS-CoV-2 in non-ICU patients demanding a randomized control clinical trial for the purpose (Huang et al., 2020). However in another study, umifenovir was shown to shorten the viral shedding interval as well as decreased the duration and cost of hospitalization for non-severe, COVID-19 patients (Wang et al., 2020d). A randomized controlled trial from Iran also highlighted that umifenovir monotherapy significantly contributes to clinical and laboratory improvements in COVID-19 patients including peripheral oxygen saturation, ICU admissions, hospitalization duration, ESR, WBC, and chest CT requirements (Nojomi et al., 2020). A randomized, double-blinded, placebo-controlled, Phase III trial is ongoing in India for the use of umifenovir to analyze the efficacy, safety, and tolerability of umifenovir in Indian COVID-19 patients (CSIR, 2020) (Kitagawa et al., 2020). Therefore, further in-depth confirmatory investigations are required to establish the efficacy of umifenovir for its dosages and durations.

##### Lopinavir and Ritonavir

Viral proteases play key roles in converting the initially translated viral protein products into the final and bioactive proteins and hence their inhibitors may serve as a potential antiviral drug. The crystallographic structure of the SARS-CoV-2 key protease (M^pro^) is observed to be highly similar to the SARS-CoV-13C-like protease (3CLpro), unlike any of the known human proteases (Báez-Santos et al., 2015). Lopinavir-Ritonavir are the viral protease inhibitors and has been observed to accelerate recovery of 10 hospitalized patients, whereas no difference was observed in a randomized trial consisting of 99 COVID-19 patients and 100 healthy subjects (Cao et al., 2020a). However, many of the patients receiving these drugs left the trial in between due to various side-effects including headache, vomiting, and diarrhea (Cao et al., 2020a). Recently, a large multicenter study has reported that early Lopinavir/ritonavir does not reduce mortality in COVID-19 patients (Lora-Tamayo et al., 2021).

##### Azithromycin

Azithromycin, a macrolide antibiotic that inhibits the bacterial protein synthesis also has some modulatory effects on the host immune cells due to its ability to shift macrophage polarization from M1 to the alternatively activated M2 phenotype. Azithromycin is shown to reduce the respiratory syncytial virus (RSV) release by inhibiting the IFN signaling and pro-inflammatory cytokine in the airway smooth muscle and epithelial cells (Traebert and Dumotier, 2005). Although a 5-day course of this antibiotic was shown to cause nearly three-fold higher cardiovascular disease-related death (Ray et al., 2012), another follow-up cohort study and independent meta-analysis of prospective randomized controlled trials reported no such risk (Baker and Couch, 2007). However, the azithromycin along with chloroquine or hydroxychloroquine is expected to have synergistic effects and hence being evaluated (Liu et al., 2020b). Recently, a nationwide Platform Randomized trial of INterventions against COVID-19 in older People (PRINCIPLE) from University of Oxford has reported that antibiotics such as azithromycin are ineffective for the treatment of such patients.

##### ACE Inhibitors and Stimulators

ACE inhibitors reduce the viral entry by the competitive inhibition of SARS-CoV-2 spike protein which binds to the host cell ACE-2 *in vitro* (Minor, 2015). Moreover, it is noted that the SARS-CoV-2 infected alveolar cells express less ACE-2 on their cell surface as compared to uninfected normal cells (Kuba et al., 2005). The knockdown of the ACE-2 expression was observed to create acute lung injury in the un-infected mice, which was histologically similar to the injuries caused by SARS-CoV-2 infection, thereby suggesting that the ACE-2 is also critical for protecting lungs (Kuba et al., 2005). These observations may suggest that patients under ACE-2 inhibitor therapy may get benefitted by stopping them while the ones not taking might get benefitted by starting these drugs. Scientists hypothesized that Non-Steroidal Anti-Inflammatory Drugs (NSAIDs), e.g. ibuprofen or RAS blockers, could exacerbate the COVID-19 by upregulating the ACE-2 which would ultimately facilitate ACE-2 and virus interaction causing the infection. Hence, in the early stages of COVID-19 outbreak, a study claimed that ibuprofen being an ACE-2 stimulator is unsafe for asymptomatic COVID-19 patients (Wan et al., 2020).

##### Convalescent Plasma

Convalescent plasma is a term referring to the pooled plasma or immuno-globulins isolated from the patients who were earlier infected in the past and then recovered from the disease. Convalescent Plasma Therapy (CPT) has been used since a long time to treat infectious diseases, such as Spanish influenza (H1N1), Avian influenza A (H5N1), SARS-CoV, and other similar viral infections and thus being presently applied for the treatment of SARS-CoV-2 infections as well (Rojas et al., 2020). Recently, one dose of 200 ml convalescent plasma was observed to immediately reduce the viral load nearly to the undetectable level and improved the oxygenation within 3 days of treatment in 10 SARS-CoV-2 positive, hypoxic, and non-intubated patients (Duan et al., 2020). Similar positive effects of CPT were observed when 200–250 ml of convalescent plasma was transfused in the five intubated patients on 10 and 22 days of admission (Shen et al., 2020). In another study, four critically ill COVID-19 patients were treated with CPT along with supportive medical care and none of the patients showed any adverse effects with treatment indicating its suitability for treatment in the current scenario (Zhang et al., 2020a). However, there are few risks, which are commonly associated with CPT including (1) Transfusion-Associated Acute Lung Injury (TRALI), (2) allergic/anaphylactic reactions, and (3) transfusion-associated circulatory overloads (TACO). Other uncommon risks include (1) infection transmission, (2) febrile non-hemolytic transfusion reactions, (3) hemolytic transfusion reactions, and (4) RBC allo-immunization (Pandey and Vyas, 2012). A meta-analysis of CPT-based clinical studies for SARS and influenza (H1N1) was reported to have no major adverse effects apart from minor fever and chills (Mair-Jenkins et al., 2015). Contrarily, a recent clinical trial has reported no significant differences in the clinical status and overall mortality of convalescent plasma treated patients compared to placebo group (NCT04383535).

Over the year, many therapeutics were repurposed and/or approved till now for the treatment of COVID-19. A few drugs being currently utilized include dexamethasone, favilavir, and remdesivir. However, a curative drug that can work on all age groups, severities (mild, moderate, and high) and patients with comorbidities is still an unmet need. Therefore, more efforts are required towards the development of a robust and effective anti-SARS-CoV-2 drug for managing the ongoing COVID-19 pandemic.

## COVID-19 Vaccines

The development of an efficient vaccine is an urgent need to control the ongoing SARS-CoV-2 pandemic and hence different stockholders including the Governments agencies, Academicians, Research and Development agencies, and private industries across the globe are putting a lot of efforts to develop the same using different vaccine platforms as described below (**Table 5**).

**Table 5:** Summary of COVID-19 vaccines in pipeline.

Platform	Target	Existing license for technology	Manufacturer	Development stage
Live attenuated vaccine	Whole virion	Yes	Codageniux/Serum Institute of India	Phase 1 NCT04619628
Inactivated vaccine	Whole virion	Yes	Sinovac (CoronaVac)	Phase 4 NCT04747821 NCT04756830
			Sinopharm/Beijing institute of Biological Sciences/Wuhan institute of Biological Sciences	Phase 3 ChiCTR2000034780 ChiCTR2000039000 NCT04612972
			Bharat Biotech International Limited with Indian Council of Medical Research and National Institute of Virology, India (Covaxin)	Phase 3 NCT04641481 and CTRI/2020/11/028976
Vector based vaccine	Spike protein	Yes	Johnson & Johnson	Phase 3 NCT04505722 ISRCTN14722499
			Gamaleya Research Institute; Health Ministry of the Russian Federation (Sputnik V) Gam-COVIDVac	Phase3 NCT04530396 NCT04564716 NCT04642339
			CanSino Biologics	Phase 3 NCT04526990 NCT04540419
			University of Oxford, AstraZeneca and Serum Institute of India (Covishield)	Phase 4 NCT04760132
Subunit vaccine	Spike protein	Yes	Novavax	Phase 3 2020-004123-16 NCT04611802
			Sanofi-Gsk	Phase ½ NCT04762680
			Clover Biopharmaceuticals	Phase 2/3 NCT04672395
			University of Queensland MF59 adjuvanted SARS-CoV-2 Sclamp vaccine	Phase 1 NCT04495933
			The State Research left of Virology and Biotechnology (EpiVacCorona)	Phase ½ NCT04527575
DNA vaccine	Spike protein	No	Inovio Pharmaceuticals	Phase 2/3 NCT04642638 ChiCTR2000040146
RNA vaccine	Spike protein	No	BioNTech/Pfizer (Comirnaty)	Phase 4 NCT04760312
			CureVac	Phase 3 NCT04674189
			Moderna (mRNA-1273)	Phase 4 NCT04760132

### Whole Virus Vaccine

One of the conventional approaches is to make use of a whole virus, which could be live attenuated or completely inactivated. Live attenuated vaccines have been highly effective in case of smallpox, chickenpox, rotavirus, and MMR infections (Minor, 2015). Resembling a natural infection so closely, this strategy can induce a quick and strong immune response but may prove to be dangerous for immunosuppressed individuals. Inactivated vaccines use killed version of the causative agent, so they do not provide immunity that is as strong as in case of live vaccines. However, this strategy has been successful for developing vaccines against Hepatitis A and seasonal flu infections (Vajo et al., 2007). A live influenza vaccine, which expresses SARS-CoV-2 proteins, has been developed by researchers in Hong Kong. A codon de-optimization technology to attenuate viruses has also been developed by Codagenix, which they plan to use as SARS-CoV-2 vaccine in association with Serum Institute of India. Chinese state-owned pharmaceutical giant, Sinopharm, in association with the Wuhan Institute of Biological Products has also developed an inactivated virus vaccine against SARS-CoV-2 (Palacios, December 11, 2020). Moreover, Beijing-based Sinovac Biotech has already completed phase 1/2 trial of its inactivated SARS-CoV-2 vaccine candidate (Gao et al., 2020c) and have initiated phase 3 trials (Zhang et al., 2020b). Bharat Biotech, an Indian vaccine developer and manufacturer, in association with Indian Council of Medical Research and National Institute of Virology, has also developed a vaccine (named COVAXIN) based on inactivated virus which has also shown promising results in phase 3 trials and recently has entered into phase 4 trials.

### Viral-Vector Based Vaccines

Such vaccines make use of a viral backbone, for example adenovirus, to introduce a SARS-CoV-2 gene into the host. Vaccines made using this strategy do not require an adjuvant to enhance immunogenicity and they promote a robust cytotoxic T cell response in order to eliminate virus-infected cells (Choi and Chang, 2013). Johnson and Johnson, a renowned player in the development of vaccine, has developed a COVID-19 vaccine using Janssen’s AdVacadenovrial vector technology, which is currently being evaluated in Phase-3 clinical trials. CanSino Biologics Inc, a china-based vaccine company, has worked on Adenovirus Type 5-based Viral Vector platform to develop a SARS-CoV-2 vaccine. The phase 1/2 trials of this vaccine are completed and CanSinoBio has already launched phase 3 trials in Mexico (Zhu et al., 2020a). Another vaccine candidate by University of Oxford, ChAdOx1 nCoV-19, is a weakened common cold virus (adenovirus) construct with the SARS-CoV-2 spike glycoprotein (S). Initial study had shown that this vaccine candidate prevents SARS-CoV-2 pneumonia in rhesus macaques (Van Doremalen et al., 2020b) and it was then taken forward for phase1/2 (Folegatti et al., 2020) and phase 2/3 clinical trials in humans and is currently in the Phase 3 trials. The Serum Institute of India has also partnered with AstraZeneca and is conducting phase 4 clinical trials for vaccine (named COVISHIELD) in different parts of the country (Wang and Teunis, 2020).

### Subunit Vaccines

Yet another class of vaccines is the subunit vaccines, which use viral proteins, most commonly in combination with an adjuvant to elicit an immune response in the host. For SARS-CoV-2, these vaccines rely mostly on preventing the binding of the virus Spike (S) protein with the host ACE-2 receptor by eliciting an immune response and generation of neutralizing antibodies in the host. Some viral surface proteins, which can be easily presented to the immune system, as potential vaccine candidates are being synthesized by the University of Queensland. Using the company’s nanoparticle technology, Novavax has also developed a stable prefusion-protein based vaccine candidate, NVX-CoV2373 and Phase 3 human trials of the same are proposed to commence soon. Additionally, Clover Biopharmaceuticals is using their Trimer-Tag technology to develop a subunit vaccine consisting of trimerized SARS-CoV-2 S-protein. French pharmaceutical group Sanofi has partnered with GlaxoSmithKline (GSK) Plc. to develop a vaccine against SARS-CoV-2, in which Sanofi will contribute the viral S-protein antigen while GSK will provide its proven pandemic adjuvant technology to the collaboration.

### Nucleic-Acid Based Vaccines

A more recent approach being adopted by many pharmaceutical companies are the nucleic-acid based platforms for vaccine development. Although, no DNA or RNA based vaccine for any viral infection has been approved for humans till date, a lot of promising candidates are coming up for SARS-CoV-2 eradication. Inovio Pharmaceuticals has developed a DNA-based vaccine in its San Diego lab, which has advance to Phase II/III trials (Smith et al., 2020). Another very promising vaccine has been developed by US based therapeutics. Their Moderna vaccine candidate, mRNA-1273, had received fast-track designation from the US FDA and is currently in the Phase 4 trials. USA drug maker Pfizer has also collaborated with a German company BNTECH, to develop a mRNA-based vaccine, BNT162 and its Phase 4 trials are ongoing and the company is now preparing to produce globally up to 50 million vaccine doses in 2020. Similarly, Curevac is also exploring mRNA vaccine platforms and has initiated Phase 2a trials of its vaccine candidate.

### Other Vaccines in Pipeline

Some non-conventional strategies are also being tried to develop a suitable vaccine to combat this virus. World’s no. 2 cigarette company British American Tobacco (BAT) has developed a vaccine using proteins from tobacco leaves and has claimed to show positive results in the pre-clinical trials. After approval from the FDA, they have planned to initiate human trials for the same. Another controversial but potential vaccine candidate is the BCG (live attenuated) vaccine used to vaccinate children against tuberculosis in some countries. University of Melbourne and Murdock Children’s Research institute, Australia had initiated a trial into the effectiveness of existing BCG vaccine in the frontline health care workers of Australia and they plan to expand this trial to include health care workers in Australia, Spain, and the Netherlands (O’Neill and Netea, 2020) and it is currently in the Phase 3 trials (Oliver, 2020).

### Latest Development of the Promising SARS-CoV-2 Vaccine Candidates

With about 10 vaccine candidates approaching the end of final stage testing, we might have a most awaited robust and effective SARS-CoV-2 vaccine very soon, but safe delivery of vaccine shots to different countries and ultimately to the pharmacies and hospitals is another challenge. A few leading companies have released the initial data of the late stage clinical trials of their vaccine candidates. The Pfizer-BioNTech vaccine (brand name: COMIRNATY), which showed 95% efficacy at preventing disease symptoms, has received emergency use authorization from the U.S. FDA for distribution of this vaccine in the U.S. (USFDA, 2020). After BNT162, Moderna’s mRNA-1273 became the second COVID-19 vaccine to receive an emergency use authorization in the U.S. (Ledford, 2020). Both these RNA vaccines have shown over 90% efficacy at preventing disease symptoms, however, the distinct composition of the lipid nanoparticle used for encasing the RNA makes the storage and shipment conditions for both of them different. While the Pfizer vaccine must be kept at −70°C, Moderna’s vaccine can be stored at −20°C for 6 months and at 4°C for about 30 days (Ledford, 2020). However, Pfizer is trying to seek permission from the FDA to store its vaccine at −25 to −15°C, temperatures more commonly found in pharmaceutical refrigerators and freezers. Moreover, the Oxford vaccine showed around 70% efficacy in the late stage trials but it could be stored and distributed at 2–8°C making it particularly more suitable for cold-chain distribution and storage worldwide (Knoll and Wonodi, 2020). Another promising vaccine candidate Sputnik V, which was the world’s first registered vaccine based on human adenoviral vector system, has shown 91.4% efficacy in the final trials and the lyophilized form of this vaccine can be easily stored at 2–8°C. Recently, COVAXIN and COVISHIELD have also been authorized to be used in India. Both the vaccines can be stored at 2–8°C and hence are in-line with the Indian needs and global market. Additionally, Johnson and Johnson’s Janssen single shot vaccine candidate has been found to be around 66% effective and it might receive emergency use authorization soon. More recently, the emergence of new mutant strains of the virus has raised concerns with respect to the efficacy of these vaccine candidates, therefore, as the world awaits a clinically approved vaccine to fight this pandemic, it is imperative that the vaccine is effective against all the strains and is easily accessible to all the countries.

## Summary

The emergence of SARS-CoV-2 from Wuhan and its transmission to the rest of the world has caused not only high morbidity and mortality but also unprecedented health and economic emergency worldwide. In the absence of a robust, extensively tested, and effective treatment regimen, the only possible option left for efficient disease management is to understand its pathogenesis and biology in detail for the development of cost-effective and efficient diagnostic systems to curtail the disease transmission and treatment regimens for controlling the disease at a population level. The scientific community has been working assiduously towards understanding the pathogen and the disease and been able to share vital information enabling rapid development of diagnostics, potential therapeutics, and vaccines to combat the disease. Whole genome sequencing of thousands of SARS-CoV-2 isolates across the world was performed to confirm and establish its potential origin, transmission, mode of infection, and ongoing evolutions. In furtherance of scientific explorations, the transmission rate of SARS-CoV-2 was reported to be higher in comparison to its ancestors, SARS-CoV and MERS-CoV, besides having high sequence similarity. The potential routes for SARS-CoV-2 transmission from animal-to-human and human-to-human *via* direct or indirect contacts were also identified which played a critical role in delaying the transmission of disease and enabling the health management system to improve on its capacity. Nucleic acid and protein-based diagnostic targets of SARS-COV-2 were identified enabling rapid development of PCR test for detecting active infection and ELISA test for detecting past-infections along with an individual’s immune response against the infection. However, both of the techniques are high-resource requiring and have a turn-around-time of ~18 h including sample processing to data analysis for reporting the results. Therefore, enormous efforts are ongoing currently to develop rapid, point-of-care, low-resource requiring, field-deployable, and cost-effective test for antigen-detection. Several FDA-approved or newly developed therapeutic molecules underwent clinical trials at a dizzying pace for rapid evaluation of their anti-SARS-COV-2 potential and as result, three therapeutics, dexamethasone, favilavir, and remdesivir, have been approved to-date to treat COVID-19 patients. Currently, advanced computational methods like AI, ML, and computer-aided drug discovery are also being explored in a coordinated manner by the biologists and clinicians to accelerate the pace of the hunt for more efficient, cost-effective, and new chemical moiety. A few vaccine candidates (such as Astra Zeneca’ COVISHIELD, Pfizer’s COMIRNATY, and Moderna’s mRNA-1273) have also shown promising results in Phase 3 clinical trials and have now entered in the Phase 4 trails, however their efficacy in preventing infection across the socio-economic spectrum of countries is still to be seen.

Therefore, based on the available information, it is becoming increasingly clear that further improvements in our understanding of pathogen and disease-biology are the key for rapid development of effective therapeutics and strategies for better management of the disease, COVID-19.

## Author Contributions

AK and NK provided the general concept. AK, RS, JK, SP, and VS drafted the initial concept of manuscript. AK, RS, JS, SP, VS, LT, and SS wrote the manuscript, and SM, SA, TS, SC, SB, and NK provided their critical comments for the manuscript. All authors contributed to the article and approved the submitted version.

## Conflict of Interest

The authors declare that the research was conducted in the absence of any commercial or financial relationships that could be construed as a potential conflict of interest.
